# PolyTB: A genomic variation map for *Mycobacterium tuberculosis*

**DOI:** 10.1016/j.tube.2014.02.005

**Published:** 2014-05

**Authors:** Francesc Coll, Mark Preston, José Afonso Guerra-Assunção, Grant Hill-Cawthorn, David Harris, João Perdigão, Miguel Viveiros, Isabel Portugal, Francis Drobniewski, Sebastien Gagneux, Judith R. Glynn, Arnab Pain, Julian Parkhill, Ruth McNerney, Nigel Martin, Taane G. Clark

**Affiliations:** a Faculty of Infectious and Tropical Diseases, London School of Hygiene & Tropical Medicine, WC1E 7HT London, UK; b Faculty of Epidemiology and Population Health, London School of Hygiene & Tropical Medicine, WC1E 7HT London, UK; c Pathogen Genomics Laboratory, King Abdullah University of Science and Technology, Thuwal, Saudi Arabia; d Sydney Emerging Infections and Biosecurity Institute and School of Public Health, Sydney, NSW 2006, Australia; e Pathogen Genomics Faculty, Wellcome Trust Sanger Institute, Hinxton, CB10 1SA Cambridge, UK; f Centro de Patogénese Molecular, Faculdade de Farmácia da Universidade de Lisboa, 1649-003 Lisboa, Portugal; g Grupo de Micobactérias, Unidade de Microbiologia Médica, Instituto de Higiene e Medicina Tropical, Universidade Nova de Lisboa, 1349-008 Lisboa, Portugal; h Centre for Immunology and Infectious Disease, Queen Mary University of London, E1 2AT London, UK; i Swiss Tropical and Public Health Institute, 4002 Basel, Switzerland; j School of Computer Science and Information Systems, Birkbeck College, WC1E 7HX London, UK

**Keywords:** *Mycobacterium tuberculosis*, Database, Genomics, Software, Molecular epidemiology, Whole-genome sequencing

## Abstract

Tuberculosis (TB) caused by *Mycobacterium tuberculosis* (Mtb) is the second major cause of death from an infectious disease worldwide. Recent advances in DNA sequencing are leading to the ability to generate whole genome information in clinical isolates of *M. tuberculosis* complex (MTBC). The identification of informative genetic variants such as phylogenetic markers and those associated with drug resistance or virulence will help barcode Mtb in the context of epidemiological, diagnostic and clinical studies. Mtb genomic datasets are increasingly available as raw sequences, which are potentially difficult and computer intensive to process, and compare across studies. Here we have processed the raw sequence data (>1500 isolates, eight studies) to compile a catalogue of SNPs (*n* = 74,039, 63% non-synonymous, 51.1% in more than one isolate, i.e. non-private), small indels (*n* = 4810) and larger structural variants (*n* = 800). We have developed the PolyTB web-based tool (http://pathogenseq.lshtm.ac.uk/polytb) to visualise the resulting variation and important meta-data (e.g. *in silico* inferred strain-types, location) within geographical map and phylogenetic views. This resource will allow researchers to identify polymorphisms within candidate genes of interest, as well as examine the genomic diversity and distribution of strains. PolyTB source code is freely available to researchers wishing to develop similar tools for their pathogen of interest.

## 1 Introduction

Tuberculosis (TB) caused by *Mycobacterium tuberculosis* (Mtb) is an important global health issue, being the second leading cause of death from an infectious disease worldwide. The World Health Organisation (WHO) has set the ambitious target of “elimination” of TB by 2050. Widespread (multi- and extensive) drug resistance and high HIV prevalence (13% of new infections) are a serious challenge to effective control [1]. There is an urgent need for better treatments and vaccines, which in turn require a deeper understanding of the biology of Mtb and epidemiology of TB disease. Knowledge of the genomic variability among Mtb isolates could result in such insights, as well as mechanisms of virulence and transmission. Human TB is caused by bacteria belonging to the *M. tuberculosis* complex (MTBC), predominantly *M. tuberculosis*, *Mycobacterium bovis* and *Mycobacterium africanum* with occasional cases of infection with *Mycobacterium caprae*, *Mycobacterium microti*, *Mycobacterium pinnipedii, Mycobacterium orygis* and *Mycobacterium canettii* reported. They are slow growing, lipid rich actinomycetales with characteristic cell walls conferring natural resistance to many antibiotics. Members of the MTBC are indistinguishable in their 16SrRNA and *rpoB* genes, recombination does not occur between strains and have approximately the same genome length; it is their host specificity what allows their differentiation [2]. It has been speculated that MTBC emerged from a common ancestor in the Horn of Africa and spread with human migrations [3], [4], [5]. Mtb is the prevailing cause of human pulmonary disease and six major global lineages have now been identified. First sequenced in 1998 [6], Mtb has a circular genome (size 4.4 Mb, GC content 65%) containing 4111 genes. No plasmids and horizontal gene transfer have been observed after the last common ancestor of MTBC [7]. The genome is characterised by limited sequence diversity resulting from a low mutation rate [8]. Insertion sequences are also responsible for genomic variation among MTBC isolates [9]. It may be said that drug treatments are driving changes in the Mtb genomes at a faster rate than any other evolutionary force [10]. In fact, polymorphisms are elevated in genes relating to antibiotic action as resistance to anti-TB drugs is caused predominantly by point mutations that arise spontaneously and are selected during unsatisfactory drug treatment. Sequential rounds of mutation and selection result in the emergence of strains resistant to multiple drugs turning TB in an even more difficult to treat disease.

Over the last two decades, molecular typing methods such as IS6110-RFLP [11], spoligotyping [12] and MIRU-VNTR [13] have been applied and revolutionised epidemiology of TB, by providing insights into the genetic diversity and population structure of MTBC [14]. Genotyping has been used extensively with epidemiological data to further understanding of TB [15]. For example, at the individual level, cases of recurrence or treatment failure can be explained in terms of reactivation with the same strain, exogenous re-infection or due to polyclonal infection [16]. At a population level, the origins and transmission dynamics of outbreaks can be determined [17], [18], [19]; whilst at a global level, TB genotypic lineages have been defined and used to monitor their geographical distribution [15]. Nevertheless, standard genotyping methods have several limitations. First, the repetitive nature of genetic polymorphisms used by molecular techniques makes them highly prone to convergent evolution [20], reducing their usefulness as phylogenetic markers. Second, the discriminative power differs between methods, meaning that results from different techniques are not always comparable [20]. Furthermore, isolates with identical DNA fingerprints have been reported to harbour significant genomic diversity [21]. Therefore standard genotyping tools, which are based on less than 1% of the genome, may not be able to accurately resolve transmission chains and distinguish disease relapse from exogenous re-infection conclusively. However, SNPs and other genetic polymorphisms derived from whole-genome sequencing (WGS) provide enough discriminatory power to assess population natural variation and predict its host–pathogen relation including virulence factors, drug susceptibility determinants and immune modulator factors with importance on the clinical manifestations [16]. Furthermore, due to its low mutation rate [19] and limited genomic diversity, the application of WGS in clinical settings is particularly effective for Mtb [22]. With the rapid decrease in DNA sequencing costs, it is foreseen that WGS will eventually be accessible and affordable enough to be an alternative to current lab-based genotyping techniques in the context of phylogenetic and epidemiological studies [18], [19], [23], [24], [25].

Given the large amount of data being generated on a routine basis from Mtb WGS projects, efforts must be focused on data analysis, accessibility, visualisation and utilisation. The TB community has a number of available web-based databases and tools to exploit the existing molecular epidemiological data [26], SNP repositories [27] and manually-annotated genomes [28]. Nevertheless, there is no tool harbouring genetic polymorphisms derived from WGS projects integrated with geographic distribution, strain type information and population structure visualisation. To fill this gap, we have developed PolyTB, a web-based tool to display Mtb genetic polymorphisms derived from publicly available WGS datasets. We compile a catalogue of SNPs, small indels and large deletions by employing the state-of-the-art variation discovery software [29]. Variants can be investigated through a genome browser reporting their chromosome coordinates, and a world map showing their global allele distribution. Additionally, the construction of phylogenetic trees based on SNPs provides an additional tool to investigate the population structure. Strain genotype information is incorporated, allowing the visualisation of associations of strain types with particular polymorphisms and/or geographical locations as well as helping correlate easily with public health epidemiological data. The integration of such data into tools like PolyTB is required to fully exploit genomic variation, and potentially boost TB control research through the discovery of new drug targets, vaccine antigens and diagnostics.

## 2 Materials and methods

Eight publicly available Mtb WGS datasets (Table 1) were downloaded from the European Nucleotide Archive (ENA) (http://www.ebi.ac.uk/ena/). All isolates (*n* = 1627) had been sequenced using Illumina paired-end technology (Illumina-GAII or HiSeq 2000), and were aligned to the H37Rv reference genome (Genbank accession number: NC_000962.3) using BWA [30]. SAMtools/BCFtools (SAMTOOLS) [31] and GATK [32] were used to call both SNPs and small indels. Variants were then selected as the intersection dataset between those obtained from both programs. Mappability values were calculated along the whole reference genome using a *k*-mer length of 50 bp and 0.04% of allowed substitutions while mapping. Non-unique SNP sites (mappability values greater than one) were filtered out. We called SNP alleles using an alternative coverage-based approach. A missing call was assigned if the total coverage at a site did not reach a minimum of 20 or whether none of the four nucleotides accounted for at least 80% of the total coverage. We plotted the sorted proportion of missing calls for all isolates and found a clear inflexion point at around 15% which was then used as a quality threshold to filter samples.

**Table 1:** Publicly available Mtb WGS datasets included in PolyTB.

Population (reference)	ENA accession number	Sample size/post-QC	Read length	Median read depth	Post-QC No. SNPs
Samara, Russia [10]	ERP000192	329/264	49	61	18,936
Midlands, UK [18]	ERP000276	390/390	75	112	19,406
Kampala, Uganda [52]	ERP000520	51/51	75	257	8021
Global key strains [4]	ERP001731	171/166	75/100	97	29,181
Bilthoven, Netherlands [19]	ERP000111	213/153	75/100	39	10,016
Vancouver, Canada [17]	SRP002589*	36/24	50	37.5	1026
Lisbon, Portugal (J. Perdigão et al., submitted for publication)	ERP002611**	84/81	100	104	6627
Karonga, Malawi (J. Guerra-Assunção et al., in preparation)	ERP000436	353/341	75	183	19,285
Overall		1, 627/1470			74,039

A set of 8 whole-genome sequencing (WGS) studies available in the public domain were downloaded from the European Nucleotide Archive (ENA). All samples were sequenced at the Wellcome Trust Sanger Institute, except * at the Simon Fraser University and ** at the King Abdullah University of Science and Technology (KAUST); all data generated using Illumina Genome Analyzer II technology, except Malawi (Karonga Study), Portugal (Lisbon) and Uganda (Kampalan Study) obtained using Illumina HiSeq 2000.

Large deletions (>100 bp) were determined using a combination of tools based on paired-end, split-read and depth of coverage approaches. In particular, Breakdancer [33], CREST [34], Pindel [35], Delly [36] and CNVnator [37] were employed followed by a *de novo* assembly-validating strategy. Reads at putative deletions (±300 bp) predicted by all five tools were extracted from bam files and subsequently *de novo* assembled using Velvet [38]. If a derived contig happened to be split into two parts when mapping it back to the reference [39], [40] with high similarity (>95%), the contig was considered a cross-junction contig (CJC) [34]. Deletions without at least one CJC were considered to be false positives and were therefore discarded. Deletions in PE/PPE genes were filtered out due to the complexity of such regions. We observed that these genes are an important source of false positives. All validated deletions were gathered and merged when having a mutual overlap greater than 95%. Secondly we kept only validated deletions sites predicted by at least two tools or occurring in at least two isolates. A summary of the bioinformatic pipeline is summarised in Supplementary Figure 1.

A set of 16 publicly available complete Mtb genomes were downloaded (Supplementary Table 1). All genomes were aligned against the H37Rv reference genome (NC_000962.3) using BWA MEM [41] and variation derived using SAMTOOLS and GATK. SNPs and small indels were then derived as the intersection dataset between those obtained from both calling software. Large deletions in complete genomes were derived with an implemented pipeline consisting of *nucmer*, *show-diff* [42] and *AGE* software [39].

Strain spoligotypes for all isolates were derived from fastQ files using *SpolPred* [43]. The best-scoring maximum likelihood phylogenetic tree was computed with RAxML v7.4.2 [44] using all 74,039 SNP sites spanning the whole genome. PolyTB has been developed using a combination of PHP, HTML, CSS, JavaScript and Perl code. The functionality in the Genome Browser page is mostly implemented by PHP code, whereas the Google Maps view employs mainly JavaScript, including the Google Maps JavaScript API. The phylogenetic analysis page required the integration of executables on the server-side and specific JavaScript libraries. A genetic distance matrix was pre-computed using PHYLIP dnadist program from all SNP sites [45]. Trees are computed on the server upon request by distance-based programs from the PHYLIP package and then displayed on the browser making use of jsPhyloSVG JavaScript library [46]. Source and data processing code is available from the corresponding author.

## 3 Results

**Figure 1:**
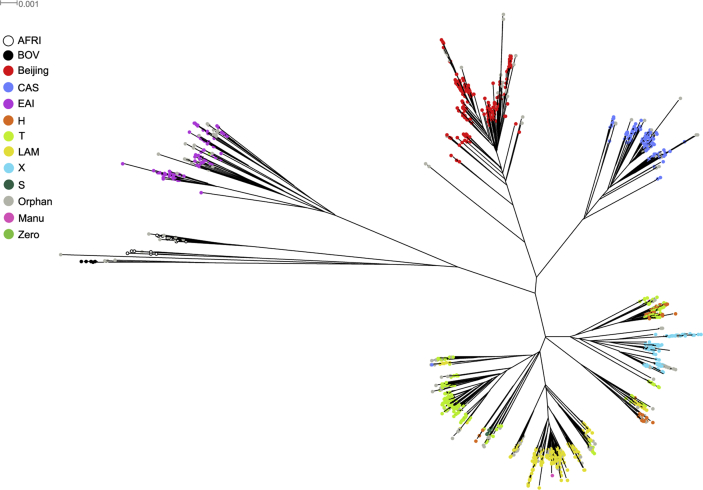
RAxML maximum likelihood phylogenetic tree constructed for all 1470 isolates (spoligotype colour-coded). Radial phylogram representation of the best-scoring maximum likelihood phylogenetic tree constructed using RAxML software. Samples are colour-coded by spoligotype strain showing a clear correlation of SNP and spoligotype clustering.

A total of 1627 Mtb isolates from 8 studies with available paired end sequence data (minimum read length 49 bp) were uniquely aligned to H37Rv (Genbank accession number: NC_000962.3) using BWA (see Methods, Supplementary 1 for a schematic of the bioinformatic pipeline). A high quality SNP dataset (*n* = 74,039) was attained by filtering the list of SAMTOOLS and GATK consensus variant calls using genomic mappability criteria. Isolates having less than 15% SNP missing calls were retained (*n* = 1470). Both the spoligotypes and lineages were inferred *in silico*, using SpolPred software [43]. All major modern MTBC lineages [47] are represented, including lineage 1 (East African-Indian (EAI) spoligotype family, 95 isolates, 6.46%), lineage 2 (Beijing, 246 isolates, 16.73%), lineage 3 (Central Asian (CAS), 170 isolates, 11.56%) and lineage 4 (715 isolates, of which 119 X, 273 T, 266 LAM, 7 S and 50 H). Ancestral lineages represented include seventeen *M. africanum* cases, 7 from lineage 5 (West African 1 family), 10 from lineage 6 (West African 2 family) and 6 cases of *M. bovis*. Nearly 15% of isolates (*n* = 218) had orphan spoligotypes, i.e. they were not previously described, but were often closely related to known spoligotypes. Figure 1 shows a radial phylogram for all samples, rooted on *M. bovis.* All major MTBC lineages are separated, with *M. bovis*, lineage 1, 2, 3, 5 and 6 isolates clustered within discrete clades, thereby demonstrating the usefulness of SNPs for strain classification. All isolates belonging to lineage 4 are grouped together, although H, T and LAM samples are dispersed among different clades as already observed [48].

To highlight the presence of site-specific lineages, edges in the tree were colour-coded by geographical location (Supplementary Figure 2). The majority of studies (7 out of 8) include isolates belonging to all genetic lineages. In contrast, samples from the Vancouver (SRP002589) study are grouped within the same clade (X spoligotype) suggesting they all resulted from the clonal expansion of the same ancestor [17]. Similarly, a well-delineated group of Beijing isolates is found to belong exclusively to the ERP000192 study carried out in Samara, Russia [10]. The geographical clustering of this sub-group of Beijing isolates corresponds to the “East European” subtype of the Beijing lineage dominant in that region.

### 3.1 Polymorphisms detected and incorporated into PolyTB

**Figure 2:**
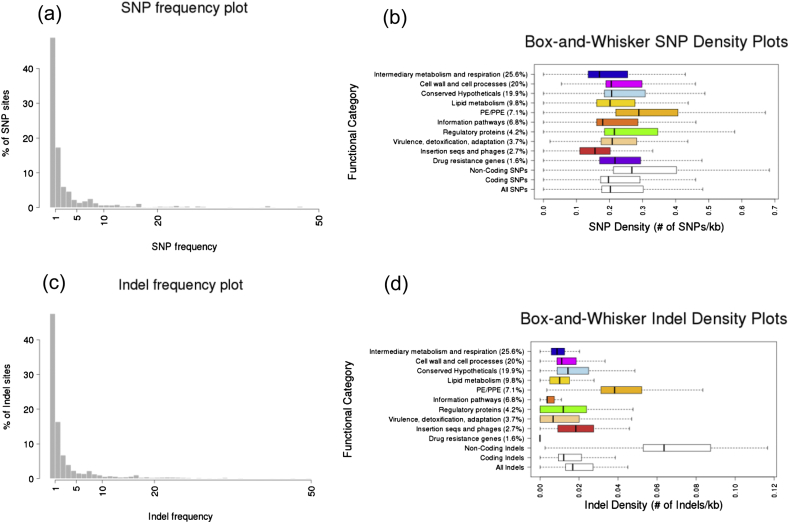
Polymorphism frequency and density plots. (a) SNP frequency bar plot; (b) SNP density plots for all, coding, non-coding and Tuberculist-functional annotated families; (c) Small indel frequency plot; (d) Indel density plots for all, coding, non-coding and functional-grouped coding indels.

Of the 74,039 high quality SNPs identified (GATK/SAMTOOLS intersection and filtered by mappability), nearly half (48.9%) were found to be private, namely observed in only one isolate (Figure 2a). In general, there were few common SNPs with only 4.6% of SNPs (*n* = 3418) present in at least 5% of samples. Most of SNPs were found in coding regions of the genome (median 88.7%) consistent with these regions comprising 91.4% of Mtb genome. The majority lead to non-synonymous (NS) changes in amino acids (median 63.0%). Overall, 1050 SNPs were found per sample on average (range 0–2261 SNPs), corresponding to a median SNP density of 1 SNP per 4.9 kb. SNP density in coding genes (median 0.20, range 0–0.50 SNPs/kb) was found to be lower than that in intergenic regions (median 0.27, range 0–0.81 SNPs/kb). Figure 2b shows the SNP density calculated across all gene functional categories as annotated in Tuberculist (http://tuberculist.epfl.ch/). As expected, the highly polymorphic PE/PPE gene families have more SNP density than the average coding regions.

We identified 4820 indel loci of size ranging between 1 and 40 bp as the intersection dataset resulting from comparing SAMTOOLS and GATK derived indels. The majority of indels are found in single isolates (47.5%) (Figure 2c). An average number of 85 small indels were detected per sample (range 0–199 indels). Both insertions and deletions accounted for an equal proportion of events, 48.8 and 51.2% respectively. Indel density was found to be five times smaller in coding genes (median of 1 indel per 83.2 kb) than in non-coding regions (median of 1 indel per 15.7 kb). As was the case with SNPs, the PE/PPE gene families have on average greater indel density than across the rest of coding regions (Figure 2d).

A total number of 800 large deletion loci (median size of 541 bp, range 100–28,862 bp) were identified after applying a combination of SV detection approaches (pair-end, split-read and depth of coverage) followed by *de novo* assembly and re-alignment validation process of candidate regions. The average number of deletions per isolate was 18 (range 0–38) corresponding to a median density of 1 deletion per 232 kb. Deletion density at coding regions, including those covering whole genes or partially, was 17 times smaller (1 deletion per 576 kb) than that calculated for non-coding regions (1 deletion per 34.3 kb).

The validity of polymorphisms was evaluated by considering known variants extracted from a set of publicly available Mtb whole genome sequences (Supplementary Table 1). A total number of 12,887 SNPs, 6749 small indel and 95 large deletion loci were identified from whole genome comparisons of 16 complete Mtb genomes against the H37Rv reference (see Materials and Methods). Our set of polymorphisms (WGS-derived variants) was compared against this validated dataset finding an overlap of 4814 SNP, 319 indel and 26 deletion loci, namely WGS-derived variant loci present in at least one of the Mtb complete genomes too. These overlapping polymorphisms were found to be more frequent (17.2%, 18.4% and 43.4% of samples for SNPs, indels and deletions) than those not shared with complete genomes (0.3%, 0.6% and 1.0%). Overall, these results indicate our set of polymorphisms encompass the known variants at the high stringency imposed in the calling procedure.

### 3.2 PolyTB and its applications

**Figure 3:**
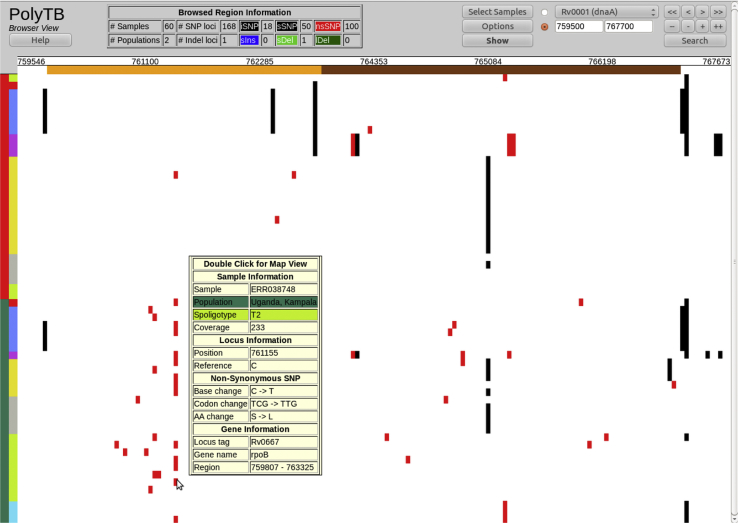
Polymorphisms at the *rpoB-rpoC* region associated with rifampicin resistance (Browser View). Genetic variants are shown at the *rpoB a*nd *rpoC* genes, loci known to be associated with rifampicin resistance. Synonymous SNPs (sSNPs) are coloured in black, non-synonymous SNPs (nsSNPs) in red and small insertions and deletions in blue and green, respectively. Cursor movement over variants displays an information box with further annotation including nucleotide, codon and amino acid changes for SNPs; and length and sequence for indels. *Locations* and *Spoligotypes* tracks are placed as colour-coded vertical bars at the left hand side of the genomic plot and provide information for samples. Sixty isolates are shown, 30 from Malawi (colour-coded in red in the Location bar) and 30 from Uganda (shown in green). Patterns of SNP difference can be observed when comparing isolates from different populations: Kampala isolates harbour many more nsSNPs at *rpoB* gene than Malawian isolates. The observed nsSNPs are likely to be the underlying cause of rifampicin resistance (Clark et al., 2013). In fact, *rpoB-*516 (A → T SNP at 761,110 bp), rpoB-526 (G → T 761,139 bp and A → G 761,140 bp) and *rpoB-*531 (C → G 761,155 bp) mutations are observed in Ugandan isolates, and correspond to nsSNPs already reported as rifampicin resistance markers [28].

PolyTB is a web-based resource (http://pathogenseq.lshtm.ac.uk/polytb) that has been designed to facilitate the exploration of MTBC genetic variation (74,039 SNPs, 4820 indels and 800 deletion sites) at a genome and global scale. The tool consists of complementary and integrated genome browser, map and phylogenetic views. The *genome browser* shows SNPs, small indels and large deletions, colour-coded and displayed at their respective genomic coordinates for the chromosome region and isolates selected by the user. Browsing options allow the user to navigate to the genes or regions of interest, with annotation tracks (top) and sample descriptions (left side) providing context for the variation. *Search functionality* has been implemented to enable the investigation of polymorphisms at genes of interest given their locus tag, functional annotation, description key words or association with anti-TB drug resistance [28]. Figure 3 shows differences on polymorphism patterns between isolates from two different populations in the neighbouring *rpoB* and *rpoC* genes, a region associated with rifampicin resistance. Known rifampicin resistance markers including rpoB-516 (corresponding to the observed 761,110 bp A → T SNP), rpoB-526 (761,139 bp G → T and 761,140 bp A → G) and rpoB-531 (761,155 bp C → G mutation) are observed in Ugandan isolates. They all correspond to NS point mutations included in diagnostic tests [49]. Across all populations there are 65 (44 NS) and 85 (56 NS) SNP loci in *rpoB* and *rpoC* genes, respectively. Users may also consider surveying genomic variants in genes with great importance for the evolution of infection and treatment outcome such as those associated with virulence, nitric oxide production and apoptosis among other possibilities.

The PolyTB markers could be considered in functional experiments or used to develop a high throughput genotypic assay for a large-scale association study of drug resistance. Overall, the browser view aims to provide a visualisation tool for the identification of differential variation patterns among isolates and populations at the same region or between different regions under study.

**Figure 4:**
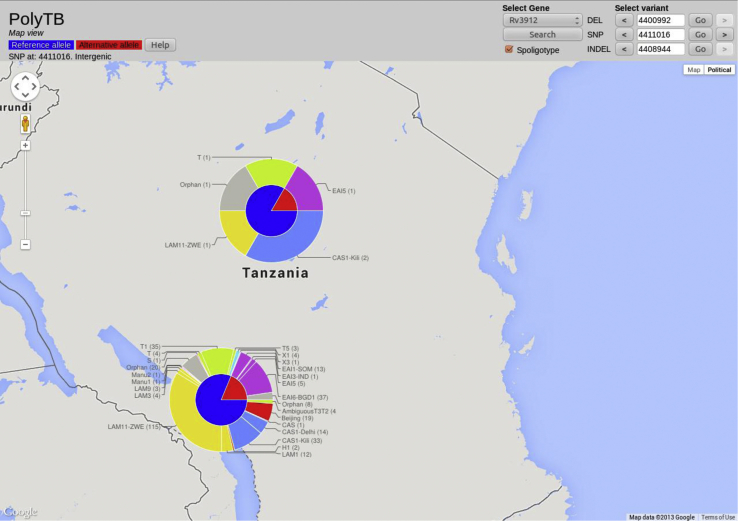
SNP associated with lineage 1 (EAI) in Tanzanian and Malawian populations (Map view). Allele frequencies are shown for the chosen polymorphic position as pie charts, either alone or combined with *in silico* inferred spoligotypes (Coll et al., 2012) to allow the visual detection of relationships between certain alleles and strain types. Reference allele frequency portions on pie charts are coloured in blue while alternative allele (i.e. non-reference) frequencies are shown in red. Outer chart portions representing relative strain type frequencies are colour-coded by main spoligotype families (AFRI, BOV, Beijing, CAS, EAI, LAM, Manu, S, T and X). In this particular case, the SNP at 4,411,016 bp position is found to be associated with lineage 1 (EAI) strains in Tanzania and Karonga (Malawi) populations, visualised as the red portion of the inner pie chart linking with the purple portions of the outer pie in both settings.

The *map view* shows the global allele distribution for a polymorphism of interest. Allelic frequencies for the chosen polymorphism are displayed as pie charts at the geographical regions from where sequenced samples were collected, either alone or combined with spoligotype frequencies as concentric pies. In the latter, outer arc-sections illustrating strain types are placed on the top of allele frequencies to visually inform of strain type associations with variants at the geographical region investigated. Figure 4 shows an informative SNP (position 4,411,016) found to be associated with lineage 1 (EAI spoligotype family) across studies (only Tanzania and Karonga (Malawi) populations shown). The main purpose of the *map view* is to provide a tool to assess the spread and frequency of WGS-derived genomic variants at a global scale as well as to enable the identification of population- and strain specific polymorphisms.

**Figure 5:**
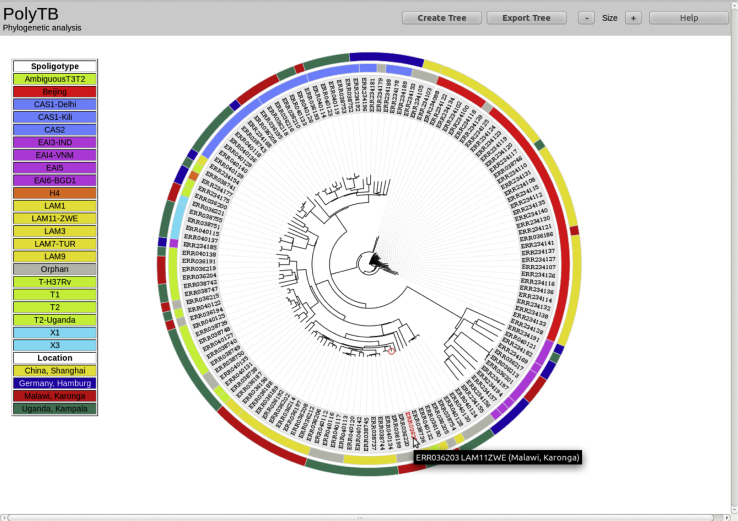
SNP-based neighbour-joining phylogenetic tree of 140 isolates belonging to four different locations (Phylogenetic view) A neighbour phylogenetic tree based on pre-calculated SNP distances is built in real time for the set of 140 isolates from Shanghai (China), Hamburg (Germany), Karonga (Malawi) and Kampala (Uganda). Spoligotype lineages and locations are colour-coded as bar charts around the tree (outer bar representing locations and the inner one spoligotypes) to enable the visual identification of correlations between spoligotype/location and phylogenetic clustering. A table summarising all colour codes will be shown at the left hand side of the page.

The *phylogenetic view* allows the user to construct phylogenies for a subset of isolates using whole-genome spanning SNPs. Spoligotypes are included to investigate whether clustering based on SNPs correlates with a strain-type. Figure 5 shows the resulting SNP-based neighbour-joining phylogenetic tree constructed for 140 isolates belonging to four different locations. Other PHYLYP distance-based methods (Fitch-Margoliash, UPGMA and Least Squares) are available too. Lineages and locations are shown as colour-coded bar charts around the tree to highlight the correlation between lineage and location with phylogenetic clustering. The aim of the phylogenetic view is to assess the genetic relatedness of isolates within and across populations as well as comparing genetic clustering with spoligotype and geographical assignation.

The PolyTB views are linked. For example, the map view is opened for a specific variant position when such a position is double-clicked on the browser view. Likewise, PolyTB is linked to external databases. Drug resistance genes were extracted from TBDreamDB [28], a database describing common mutations associated with drug resistance in Mtb. Furthermore, if a particular gene is double-clicked on the top annotation track in the browser view, the user will be forward to its Tuberculist (http://tuberculist.epfl.ch/) entry page containing further annotation information.

## 4 Discussion

Rapid, low-cost genome sequencing is expected to have a huge impact on molecular epidemiology, enabling researchers to gain insights at patient, community and global levels [16], [18], [19]. In addition to tracking the spread of pathogens, WGS is proving to be a valuable tool to carry out microbial evolution studies over different time and space scales, within a single patient over months or years; or globally across centuries [50]. Given the cost decline witnessed in recent years [23], it is foreseen that WGS will eventually be the technology of choice in clinical settings, and for epidemiological and evolutionary studies [16], [19].

Although the TB community have available web-based databases to exploit the existing genotyping data for MTBC [51], there is no such tool gathering the increasing amount of genetic polymorphisms derived from WGS projects [27]. Given the magnitude of the genomic data being generated on a routine basis, efforts must be focused on analysing and presenting this data in a robust and useful manner for the research and public health communities. In this sense, the present release of PolyTB makes it the largest open-access repository of genetic polymorphisms derived from WGS projects. For example, we include over ∼70 k SNPs (51.1% observed in more than one isolate), at least 10-fold more than any other database. The expandable database goes beyond SNPs, and includes small indels and large deletions derived by employing the state-of-the-art variation discovery software. Robust quality control and standardised procedures applied across samples ensures that the datasets are directly comparable. Overall we found that small indel and large deletion densities in coding genes were 5 and 17 times smaller than in non-coding regions respectively. This considerable reduction in polymorphism density at coding regions can be explained in terms of the potential deleterious effects of these variants in the genome, leading to their selective removal by purifying selection.

PolyTB consists of three complementary and integrated views with links to other TB databases [27]. The browser view will allow researchers to investigate genomic diversity within regions or genes of interest, such as host-infection and drug resistance related loci, as well as visualise differential variation patterns among isolates at the same region or between different regions under study. Although Mtb strains were historically confined to their endemic geographical locations, migration has led to a more global distribution. Modern modes of transport mean that TB is now easily spread across regions and continents. It is possible to monitor the spread of Mtb lineages through phylogenetic markers as well as track drug resistance markers, which emerge *de novo* and independently of strains, with a discriminatory power never achieved before. In this context, the map view provides a tool for the epidemiological surveillance of tuberculosis through the geographic distribution of strains and clinically important genetic variants, such as those driving drug resistance. Indeed, knowledge of transmission across lineages and continents is essential to those who need to devise national prevention and control programmes. Similarly, the main purpose of the phylogenetic view is to assess the genetic relatedness of isolates within and across studies as well as comparing genetic clustering with traditional spoligotypes and lineages.

Ongoing work is investigating strain-specific polymorphisms, variants associated with drug resistance, and the *de novo* assembly of poorly characterised regions, including those related to host-immunology (e.g. PE and PPE families). These results, new datasets, provision of meta data such as phenotypes, and novel SV discovery pipelines will be incorporated into future versions of the tool.

Overall, the high global burden of TB requires new control insights from the increasing number of Mtb WGS studies. Knowledge of the genetic diversity across populations, among other factors, will assist in the understanding of Mtb biology, required to develop new drugs and novel vaccines. In this context, PolyTB aims to bring together all existing genomic diversity into an integrated database and make it available for the TB community. The underlying source code is also available increasing the utility amongst researchers aiming to build similar sites for their pathogen of interest.

## Ethical approval

Not required.

## Funding

This work was supported by a Bloomsbury Research Fund PhD studentship.

## Conflicts of interests

None declared.
