# Supplementary material for: PolyTB: A genomic variation map for Mycobacterium tuberculosis
Source: Tuberculosis (Edinb). 2014 May;94(3):346–54. doi: 10.1016/j.tube.2014.02.005 (PMC4066953; doi:10.1016/j.tube.2014.02.005)
Supplement: Supplementary file 1 [file mmc1.pdf]

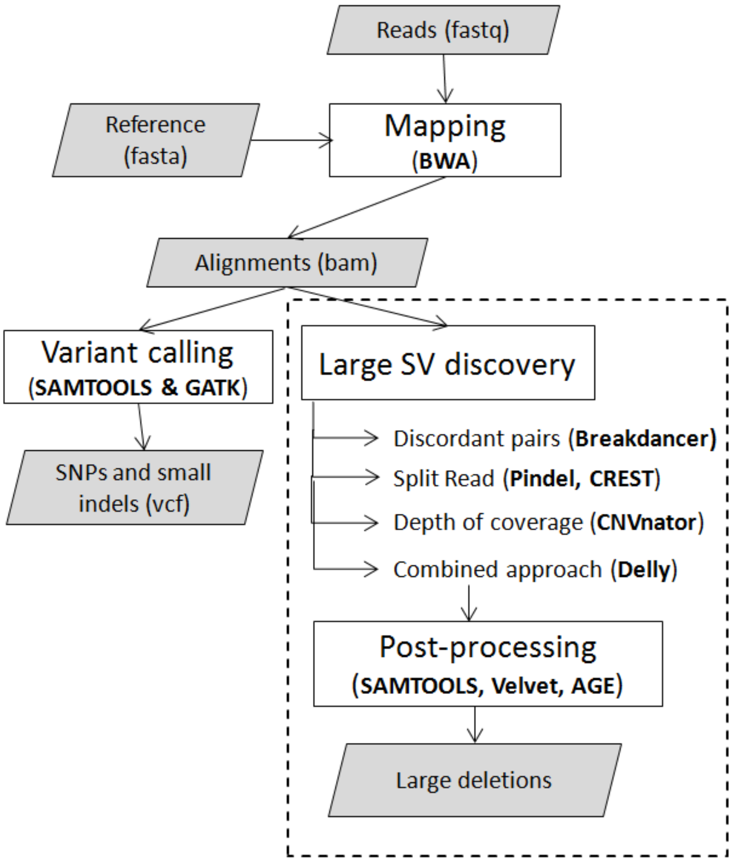

Supplementary Figure 1 Genomic variation discovery pipeline

Schematic of the genomic discovery pipeline implemented to compile a catalogue of SNPs, small indels and large deletions from Illumina paired-end sequence data. Raw reads are initially mapped to the reference genome H37Rv using BWA and the resulting BAM files used as input to subsequent software. SNPs and small indels were derived using SAMTOOLS and GATK. Large deletions were determined employing a combination of SV detection tools based on different approaches (pair-end, split-read and depth of coverage) followed by *de novo* assembly and re-alignment validation process of candidate regions.
