# Supplementary material for: PolyTB: A genomic variation map for Mycobacterium tuberculosis
Source: Tuberculosis (Edinb). 2014 May;94(3):346–54. doi: 10.1016/j.tube.2014.02.005 (PMC4066953; doi:10.1016/j.tube.2014.02.005)
Supplement: Supplementary file 2 [file mmc2.pdf]

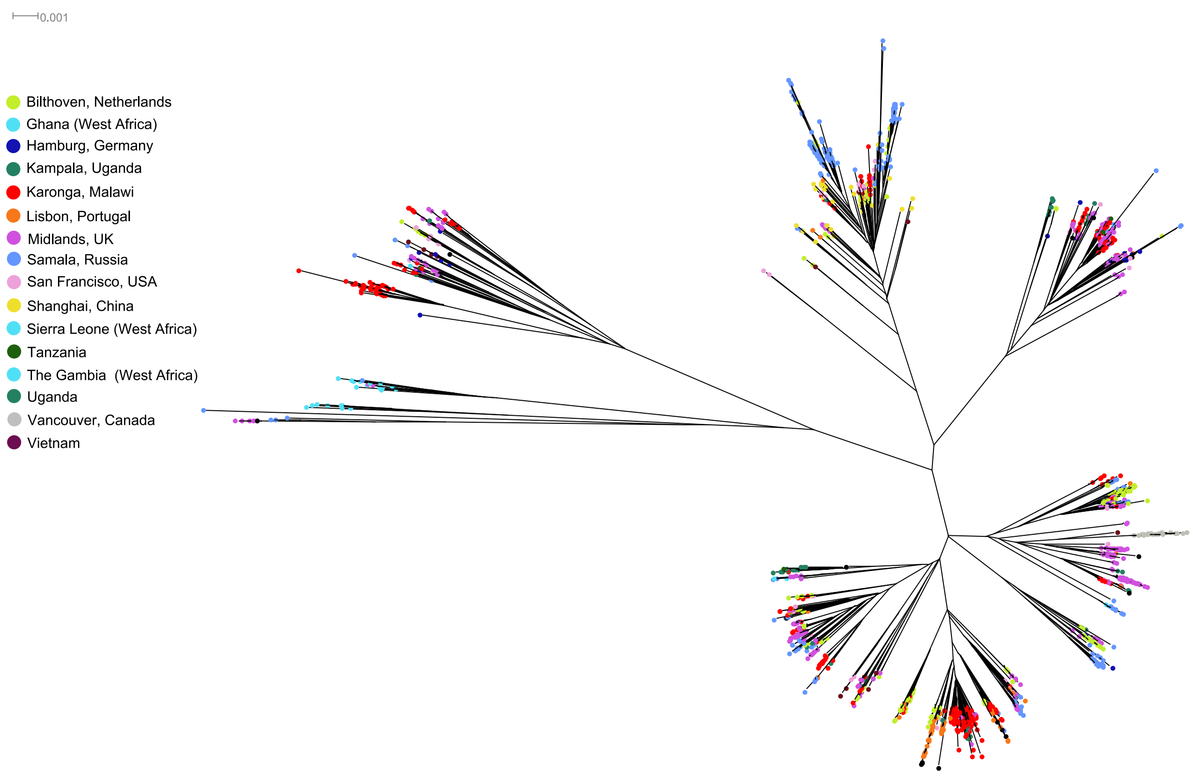

Supplementary Figure 2 RAxML maximum likelihood phylogenetic tree built for all 1,470 isolates (colour-coded by geographical location)

Radial phylogram representation of the best-scoring maximum likelihood phylogenetic tree constructed using RAxML software. Samples are colour-coded by geographical location to highlight the presence of site specific strains.
