# Supplementary material for: PolyTB: A genomic variation map for Mycobacterium tuberculosis
Source: Tuberculosis (Edinb). 2014 May;94(3):346–54. doi: 10.1016/j.tube.2014.02.005 (PMC4066953; doi:10.1016/j.tube.2014.02.005)
Supplement: Supplementary file 3 [file mmc3.docx]

Supplementary Table 1 Mtb complete genomes used to generate a set of validated SNP, indel and large deletion loci.

| Genome name (Genbank Accession Number) | SNPs (n) | SNPs  (% observed) | Indels (n) | Indels  (% observed) | L.arge Del. (n) | L.arge Del.  (% observed) |
| --- | --- | --- | --- | --- | --- | --- |
| 7199-99 (NC_020089) | 1,013 | 72.75 | 108 | 70.37 | 12 | 41.67 |
| CAS/NITR204 (NC_021193) | 4,620 | 23.79 | 1,963 | 4.12 | 2 | 100 |
| CCDC5079 (NC_017523) | 2,131 | 60.39 | 385 | 25.97 | 11 | 54.54 |
| CCDC5180 (NC_017522) | 1,751 | 71.50 | 185 | 55.67 | 13 | 46.15 |
| CDC1551 (NC_002755) | 1,222 | 66.77 | 207 | 36.71 | 15 | 46.67 |
| CTRI-2 (CP002992) | 983 | 79.14 | 99 | 69.70 | 14 | 42.86 |
| EAI5/NITR206 (NC_021194) | 2,187 | 58.71 | 251 | 29.08 | 11 | 72.73 |
| F11 (NC_009565) | 986 | 80.16 | 92 | 75.00 | 8 | 37.50 |
| KZN 1435 (NC_012943) | 1,005 | 78.50 | 113 | 55.75 | 7 | 42.86 |
| KZN 4207 (NC_016768) | 994 | 79.48 | 93 | 67.74 | 6 | 66.67 |
| KZN 605 (NC_018078) | 1,019 | 78.02 | 112 | 56.25 | 6 | 50.00 |
| RGTB327 (CP003233) | 1,145 | 56.33 | 1,821 | 2.58 | 7 | 0.00 |
| RGTB423 (NC_017528) | 2,615 | 71.59 | 2,027 | 4.53 | 4 | 0.00 |
| Beijing/NITR203 (NC_021054) | 2,338 | 56.97 | 198 | 39.89 | 0 | 0.00 |
| Erdman (AP012340) | 1,159 | 70.06 | 114 | 61.40 | 25 | 32.00 |
| UT205 (NC016934) | 808 | 79.83 | 94 | 62.76 | 13 | 69.23 |
| Overall | 12,887 |  | 6,749 |  | 95 |  |

Summary of genetic variation extracted for a set of 16 Mtb complete genomes downloaded from Genbank. Genetic variation across all 16 genomes was derived with respect to the H37Rv reference genome (Genbank accession number NC_000962.3). The number of SNPs, indels and large deletions per genome are shown in columns 2, 4 and 6 respectively. The percentage of variants also observed in the WGS public dataset (namely present in at least one of the 1,470 isolates included in this study) are indicated in columns 3, 5 and 7. Overall values represent the total number of variant sites discovered across all 16 genomes.
